# Cytosine-Rich DNA Fragments Covalently Bound to Carbon Nanotube as Factors Triggering Doxorubicin Release at Acidic pH. A Molecular Dynamics Study

**DOI:** 10.3390/ijms22168466

**Published:** 2021-08-06

**Authors:** Pawel Wolski, Krzysztof Nieszporek, Tomasz Panczyk

**Affiliations:** 1 Jerzy Haber Institute of Catalysis and Surface Chemistry, Polish Academy of Sciences, ul. Niezapominajek 8, 30239 Cracow, Poland; pawel.wolski@ikifp.edu.pl; 2 Department of Theoretical Chemistry, Institute of Chemical Sciences, Faculty of Chemistry, Maria Curie-Sklodowska University in Lublin, pl. Maria Curie-Sklodowska 3, 20031 Lublin, Poland; krzysn@hektor.umcs.lublin.pl

**Keywords:** carbon nanotube, i-motif, cytosine-rich DNA, doxorubicin, drug delivery, pH change

## Abstract

This works deals with analysis of properties of a carbon nanotube, the tips of which were functionalized by short cytosine-rich fragments of ssDNA. That object is aimed to work as a platform for storage and controlled release of doxorubicin in response to pH changes. We found that at neutral pH, doxorubicin molecules can be intercalated between the ssDNA fragments, and formation of such knots can effectively block other doxorubicin molecules, encapsulated in the nanotube interior, against release to the bulk. Because at the neutral pH, the ssDNA fragments are in form of random coils, the intercalation of doxorubicin is strong. At acidic pH, the ssDNA fragments undergo folding into i-motifs, and this leads to significant reduction of the interaction strength between doxorubicin and other components of the system. Thus, the drug molecules can be released to the bulk at acidic pH. The above conclusions concerning the storage/release mechanism of doxorubicin were drawn from the observation of molecular dynamics trajectories of the systems as well as from analysis of various components of pair interaction energies.

## 1. Introduction

Biomedical applications of carbon nanotubes, CNT, have been considered in literature for many years mainly in the context of tissue engineering, sensing, diagnostic and finally as therapeutic or teranostic agents [1,2,3]. However, pristine carbon nanotubes need additional treatment in order to tune their biocompatibility, reduce toxicity or produce specific chemical properties by their covalent or non-covalent functionalization [4,5,6]. The interesting examples are carbon nanotube—DNA constructs analyzed as drug or gene delivery systems [7,8,9,10,11,12]. Among many papers discussing promising properties of such systems particularly interesting are studies related to telomeric DNA fragments interacting with carbon nanotubes. It was namely found that carbon nanotubes stabilize some of noncanonical DNA structures like i-motifs [13,14,15]. 

Application of cytosine-rich telomeric fragments of DNA as components of drugs carriers has been described in several studies [16,17,18,19,20,21,22]. This is because such oligonucleotides reveal interesting properties of reversible folding/unfolding in response to pH change. According to literature data cytosine-rich ssDNA fragments with sequences (CCCTAA)_3_CCCT (which we shortly denote as iM) can fold into i-motif spatial form at pH slightly lower than 5 [23]. Then, half of the cytosines undergo protonation and extra hydrogen bonds appear between protonated and native non protonated cytosines. The i-motif can unfold into a hairpin or random coil [24] spontaneously after bringing pH to ca. 7 and the folding/unfolding transition can be repeated again by switching the pH of solution accordingly. 

The described changes of pH are natural phenomena occurring in a tumor microenvironment. It is well known that the tumor microenvironment exhibits a reduced pH as a result of anaerobic glycolysis. This is caused by the accumulation of protons in the extracellular matrix, which are by-products of a rapid, oxygen-deficient metabolism [25,26]. Moreover, many molecules or ligands are internalized by cells according to the endocytic cycle and end up in the endosomes, which are intracellular organelles revealing pH lower than 5 [27]. Therefore, the reversible folding/unfolding of cytosine-rich DNA fragments can be utilized in processes of drug unloading precisely in cancer cells. 

As mentioned, the cytosine-rich DNA fragments were studied, in conjunction with other moieties, as pH controlled molecular switches. Chen et al. utilized silica mesoporous materials as storage space with corks formed by gold coated cytosine-rich DNA fragments [16,17]. Song et.al. [18] proposed a model with gold nanoparticle covered by dsDNA fragments containing cytosine-rich sequences and found it as an effective carrier of doxorubicin. Similarly, Xu et.al. [19] proposed a model of doxorubicin carrier based on dsDNA fragments and still consisting of the iM sequences. In another study, Kim et.al. [20] utilized iM modified exosomes as platforms for doxorubicin (DOX) storage and release with iM folding/unfolding property as a key factor for controlled release of DOX. Intercalation of DOX within dsDNA fragments attached to gold nanoparticles was studied in the work by Park et.al. [21]. Hu and Niemeyer proposed a novel hydrogel material composed of silica nanoparticles, CNT and DNA fragments rich in CG/GC sequences and showed its ability to deliver DOX molecules to HeLa cells [28]. Therefore, we can conclude from that short literature review that mainly silica or gold nanoparticles are considered as ‘cores’ of drug carriers utilizing the folding/unfolding property of iM sequences. Moreover, the iM parts are usually considered in conjunction with complementary strands forming dsDNA fragments, which are able to disintegrate at the reduced pH. We can also note that the proposed mechanisms of actions are usually rather complex and sometimes involve multiple stages/reactions.

In one of our recent papers, we analyzed and discussed the properties of a system composed of a carbon nanotube and co-adsorbed DOX and iM sequences [22]. That model revealed that binding of iM to carbon nanotube surface depends strongly on the presence of DOX and folding/unfolding of iM. Unfolded iM was able to co-adsorb with DOX on the CNT surface strongly and the adsorption was strongly weakened after folding of iM into i-motif form. It needs to be underlined that the iM chains were not covalently bound to the CNT surface, but they were moving freely on the surface.

Here, we propose a new concept of drug carrier which still involves folding/unfolding property of iM but its architecture is conceptually simple. We are also trying to link useful properties of carbon nanotubes [6] and iM sequences in a single functional device. Moreover, our methodology relies on direct analysis of mechanism of action on the molecular level.

Thus, the aim of this work is analysis of (covalently) i-motif functionalized carbon nanotubes as pH controlled carriers of DOX. First, we consider a mechanism of encapsulation of drug molecules in the internal space of CNT and analyze ability of blocking of DOX against spontaneous escape by i-motifs chains at neutral pH. The outcomes from this part of study focused our attention on another possible mechanism of DOX immobilization. That is intercalation of drug molecules between i-motif chains linked to the CNT tips. This mechanism is carefully analyzed since it offers promising results from relatively simple molecular constructs. 

## 2. Results and Discussion

### 2.1. Definitions of the Analyzed Systems

Let us carefully define which system types we were dealing with. Generally, we were considering a single walled carbon nanotube with chirality (20.0), which was covalently functionalized by cytosine-rich DNA fragments, iM, on the tips. The assumed chirality generates single-walled CNT with the diameter ca. 15 Å, which is enough for accommodation of DOX molecule in the inner cavity of the CNT. We studied two nanotube lengths: 100 Å and 20 Å and the reasoning for application of a very short 20 Å CNT will be provided soon. The number of DNA chains was 2 or 4 on each side of the CNT. Additionally, we studied the systems at two pH cases: neutral and acidic ones which should be understood as lower than ca. 5. A combination of the mentioned parameters gives us seven different systems, which are defined in Table 1. 

The systems with names starting from “E” were designed as nanocontainers storing DOX molecules in the inner cavity of the CNT, while the systems with names starting from “I” utilize the concept of intercalation of drug molecules within the bundles of iM chains localized at the CNT tips. In both cases, the covalent linkage of the DNA fragments to the CNT tips was assumed, thus the next subsection explains how that step was done in terms of force field topology and molecular architecture. 

### 2.2. Covalent Functionalization of CNT by DNA Motifs

**Figure 1:**
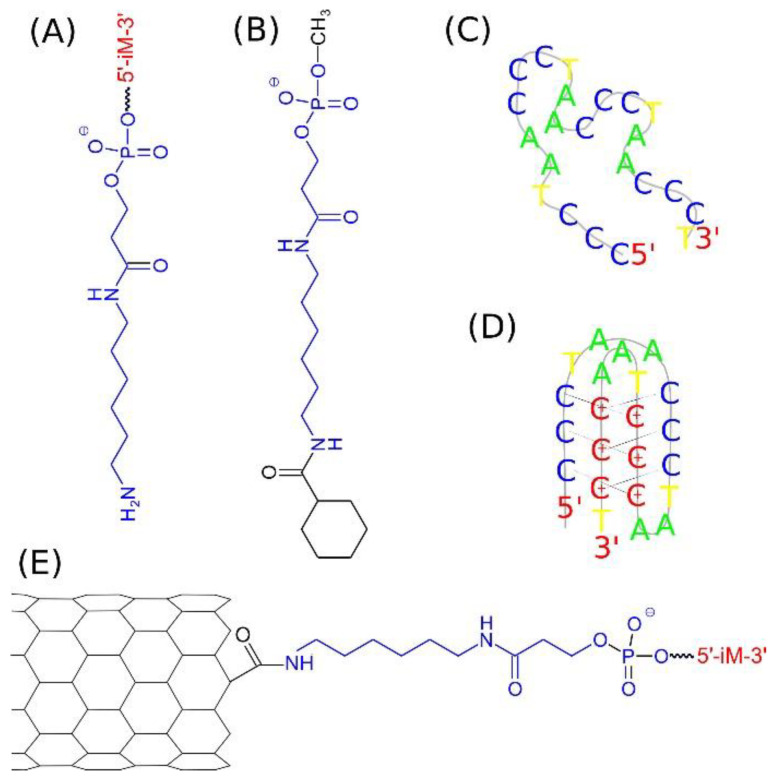
Essential components of the analyzed systems. (**A**) amino DNA linker composed of 5′ amino C6 linker (blue) and iM sequence (red); (**B**) template molecule of the amino C6 linker used for computation of atomic partial charges; (**C**) random coil of the DNA iM sequence at neutral pH; (**D**) iM sequence folded into i-motif structure at acidic pH; (**E**) on tip functionalized carbon nanotube by amino DNA linker with the iM sequence.

The nanotube ends (tips) formally contain carbon atoms saturated by hydrogens or other chemical fragments depending on the assumed chemical treatment. Typical purification procedures of carbon nanotubes involve oxidation by strong acids which leads to carboxyl groups formation in defect sites and particularly on the CNT ends which simultaneously leads to their opening [29]. The presence of carboxyl groups on the CNT tips allows for covalent linkage of many functional groups including DNA strands. Typical procedure utilize *N*-ethyl-*N*-(3-dimethylaminopropyl) carbodiimide hydrochloride (EDC) as coupling agent [30], which activates the carboxyl groups and allows for substitution of primary amine oligo DNA linkers. In our study, we selected for that purpose the 5′ amino C6 linker, which is available commercially, for generation of the end product, that is DNA functionalized CNT. The chemical structure of 5′ amino C6 linker and scheme of the DNA functionalized CNT is shown in Figure 1.

The scheme shown in Figure 1A represents the structure of the amino C6 linker with the DNA sequence iM bound to the phosphate residue. The iM sequence, composed of 22 nucleic bases, is representative of the telomeric cytosine rich strand 5′ (CCCTAA)_3_CCCT3′. That sequence can take at least two different spatial forms: random coil, Figure 1C, at the neutral pH and i-motif, Figure 1D, at the acidic pH. The amino linker can be covalently bound to the carboxyl functionalized carbon nanotube tip forming peptide bond. In that way the iM sequence can be linked to the nanotube tip leading to the structure in Figure 1E. As mentioned, we analyzed carbon nanotubes with two or four linkers attached to each CNT side, so the structure in Figure 1E represents only a schematic view with only one linker attached.

The property of iM sequence, that is the ability of reversible folding into i-motif structure at acidic pH and unfolding at the neutral pH [23], leads to consideration of two cases. Namely, the random coil form of iM (Figure 1C), which is representative to the neutral pH and i-motif form of iM (Figure 1D), which appears at acidic pH when half of cytosines within the iM sequence become protonated. This strongly affects either the atomic topology of the system or the force field parameters. Thus, the systems listed in Table 1 were generated by manual combination of cartesian coordinates of CNT, linkers and iM forms and using such a rough pdb file as input to LEaP program from AmberTools16 package [31]. 

### 2.3. Encapsulation and Release of DOX from CNT Interior

In our earlier studies, we found that DOX can be encapsulated in the internal space of (30.0) bare carbon nanotube. However, the encapsulation turned out to be unstable with the free energy of spontaneous DOX release of ca. 22 kJ mol^−1^ [32]. In other studies we analyzed the case of PEGylated CNT, which should be considered as a more realistic case since bare CNTs are highly hydrophobic and cannot be directly used as drugs carriers. That more advanced model revealed that PEG chains readily incorporated to the nanotube interiors and formed corks which acted as barriers preventing DOX molecules from the release either at neutral or at acidic pH [33]. Therefore, we still continued a search for an efficient blocker of DOX in the CNT interior at neutral pH and only weak (or without that property) modifier of DOX interaction with CNT at acidic pH. 

The property of iM chains that is reversible folding into i-motif and unfolding into random coil in response to pH change is thus very promising. This is because the iM chains should, in principle, act similarly to PEG chains at neutral pH, i.e., form corks/knots at the CNT ends and remove them at acidic pH due to their strong affinity to formation of spatial structure of i-motif. 

The key factor in construction of a drug carrier is stable attachment of drug molecules to the carrier at normal conditions, i.e., during the storage or transportation. Of course, the carrier should release the drug after reaching the target site or in response to a triggering factor, but definitely the first condition is necessary. Thus, in order to verify whether iM functionalized CNT is able to store DOX in the internal space of CNT, we build the systems E4D20, E4D40 and E2D40. In these systems, the CNT has four or two iM chains in each side and is filled in with 20 DOX molecules. In the first stage of simulations, DOX molecules were kept inside the CNT due to application of external forces (springs) because the initial strain could lead to artificial flow of DOX molecules. Therefore, after 500 ps of the equilibration with the external constraints, the springs were removed and the systems evolved naturally.

**Figure 2:**
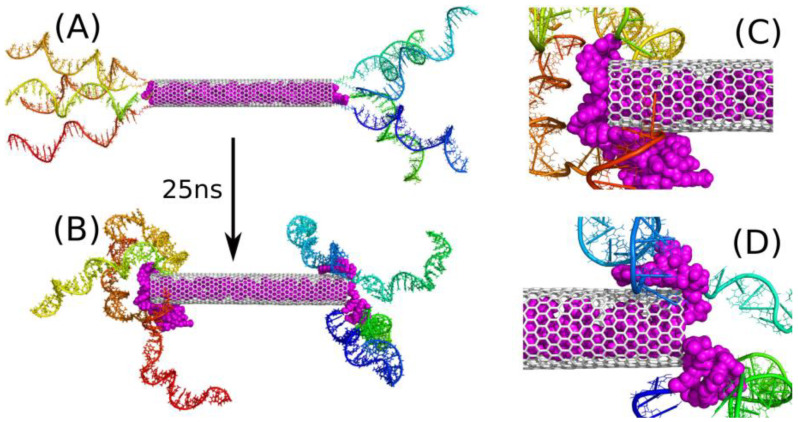
(**A**) Initial state of E4D20 system, i.e., with all DOX (magenta) molecules encapsulated in the CNT interior; (**B**) the state of E4D20 system after 25 ns of simulations. Zoomed-in sections of (B) showing the left (**C**) and right (**D**) end of the CNT with the released DOX molecules. The rainbow colors of iM chains are for illustration purposes only.

Let us first analyze the behavior of E4D20 system. Figure 2 shows the initial structure of this system (A) and the final one (B), after 25 ns of simulations. The initial structure was build semi-manually because the system is huge, and it was reasonable to start from a structure that is possibly close to the desired one. As we expected that iM chains would form knots near the nanotube ends, the initial state was generated by putting successive iM chains axially and with possibly small separation between them. Additionally, 20 DOX molecules were placed inside the CNT in a way preventing them from overlapping. This step was completed by relaxation runs in which the iM chains were, similarly to DOX molecules, pinned to their initial positions by external springs. Thus, the state in Figure 2A represent a somehow artificially constructed state, but this state is fully relaxed and there are no extra forces which could propel iM chains or DOX molecules unnaturally. The state in Figure 2B is obtained after 25 ns of MD simulations without any external constraints applied. This is not very long simulation time, but it is enough to draw essential conclusions concerning the property of the drug carrier. 

The conclusions drawn from the analysis of Figure 1B are clear: DOX molecules are not blocked in the CNT interior by iM chains. After 25 ns of simulation we observed spontaneous release of a few DOX molecules from the nanotube (magenta spheres in Figure 2). The release was quick due to high initial concentration of DOX within the nanotube, which was kept constant during the equilibration stage by the application of external springs. The released molecules started to interact with iM chains immediately, which led to reduction of their mobility and intercalation. As a result, no other DOX molecules were able to escape from the nanotube within the studied 25 ns. On the other hand, the release of DOX is an activated process, as found in ref. [32], with the energy barrier of ca. 22 kJ mol^−1^ for a wider (30.0) nanotube. In the case of a (20.0) nanotube, the activation barrier is probably bigger or similar. Nevertheless, the activation barrier greatly reduces the release rate of DOX, and in the considered simulation time of 25 ns, observation of such an event is rather unlikely.

The state in Figure 2B shows that the iM chains tend to separate from each other in the bulk and approach the nanotube surface. A similar phenomenon was also observed in our earlier study [22] concerning interaction of free telomeric cytosine-rich DNA fragments with surfaces of carbon nanotubes. It turned out that these DNA fragments rather weakly adsorb on the CNT surface and are able to detach from the surface and exist in the bulk as free molecules. However, the presence of DOX led to significant stabilization of adsorption of such a mixture on the CNT surface. Here, we also see (Figure 2C,D) that the triple interaction of DOX-CNT-iM occurs first and is strong enough to prevent DOX from escaping immediately into solution.

**Figure 3:**
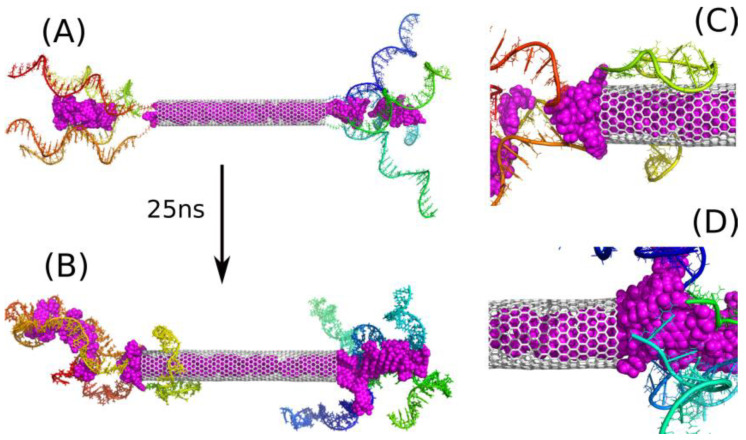
(**A**) Initial state of E4D40 system, i.e., with additional 20 DOX (magenta) molecules placed initially in the space between iM chains; (**B**) the state of E4D40 system after 25 ns of simulations. (**C**) and (**D**) show zoomed-in areas around CNT tips. The rainbow colors of iM chains are for illustration purposes only.

As seen in Figure 2B DOX molecules do not detach from the carrier, but are bound to iM fragments. The escape of more DOX from the CNT interior was found as a very slow process and it was rather impractical to continue calculations until more DOX molecules escape from the CNT and show their final localization within the carrier structure. As already mentioned the free energy barrier preventing DOX from the escape from (30.0) bare CNT was ca. 22 kJ mol^−1^ therefore the probability of observation of such an event in the narrower nanotube is very small within the applied simulation timescale. Taking into account the macroscopic timescale, a barrier of 20–50 kJ mol^−1^ means that the state is unstable at ambient temperatures and DOX molecules spontaneously escape from the CNT interior. Thus, the trapping of the released DOX molecules by flanking iM chains is a very important observation, since stabilization of iM fragments, when they form a mixture with DOX as found recently [22], offers a promising strategy of DOX immobilization within the carrier structure. Therefore, we continued the analysis with a modified computational setup. That is, we started calculations from the same state as in Figure 2A, but additional 20 DOX molecules were incorporated into the space between the iM chains. Thus, we obtained the state shown in Figure 3A and continued calculations for 25 ns reaching the state shown in Figure 3B. 

The addition of those 20 extra DOX molecules simulates the state that should roughly appear after a very long time due to activated release of DOX from the nanotube. Of course, the positions of those new molecules are not identical to possible positions reached by spontaneous motion, but this is not a critical point. The most important thing is that those additional 20 DOX molecules did not go to the bulk, but became trapped between iM chains as well. Additionally, several DOX molecules from the CNT moved beyond the nanotube and formed kinds of ‘corks’ together with those 20 extra DOX molecules in the vicinity of the CNT tips. These ‘corks’ turned out to be very stable, and they blocked other DOX molecules from the escape from the CNT interior. 

Thus, the strength of binding of DOX molecules in the iM bundles is the decisive factor concerning the ability of the considered structure to function as a DOX carrier. However, direct determination of the free energy of binding is technically very difficult due to the size of the system and huge computational costs. Nevertheless, in our recent work, we analyzed the work required for detachment of a DOX molecule from a single iM random coil and we found that this work is ca. 150 kJ mol^−1^, which is enough for stable binding at normal conditions [22]. Of course, the model studied in ref. [22] is much simpler than the current case, but we can expect that the binding in a more complex configuration will be even stronger. We therefore conclude that the studied systems E4D20 or E4D40 can work as platforms for storage or transportation of DOX. In the case of E4D20 we should obtain the same architecture like shown in Figure 3B, but more time is needed for partial release of a sufficient number of DOX molecules finally forming non-permeable corks for other molecules still residing in the CNT interior.

Further analysis of Figure 3B leads to the conclusion that the number of iM chains applied seems to be excess. Clearly at least one or two chains do not take part in interaction with DOX and they rather tend to wrap the CNT external surface. This effect can be useful because the iM chains are hydrophilic and biocompatible, so creation of such a masking layer on the CNT sidewall could be an additional advantage. However, let us skip at the moment surface modification of carbon nanotubes and focus on the encapsulation and blocking of drug molecules. 

**Figure 4:**
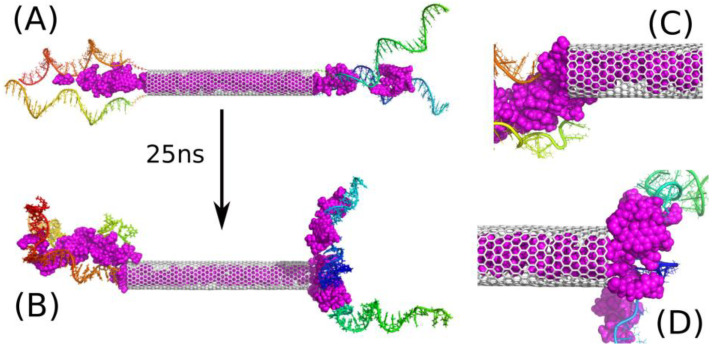
(**A**) Initial state of E2D40 system, i.e., with additional 20 DOX (magenta) molecules placed initially in the space between iM chains and with only two iM chains in each side; (**B**) the state of E2D40 system after 25 ns of simulations. (**C**,**D**) show zoomed-in areas around CNT tips.

Figure 4 shows analogous results like Figure 3, but in this case, half of iM chains was removed so there is only two iM chains on each CNT side. The state of such a system after 25 ns of simulation is shown in Figure 4B, and its analysis leads to the conclusion that two iM chains are indeed enough for trapping DOX molecules between them. Of course, this is valid for the particular type of the nanotube. If we use much wider and/or multi-walled nanotube, the distance between iM chains could be too large and the ‘zipper’ function of DOX plus two iM chains would be disabled. 

**Figure 5:**
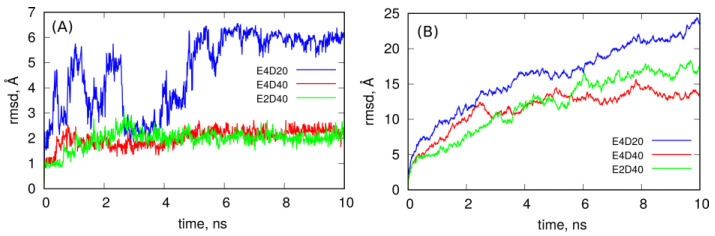
(**A**) Root of mean squared displacement, rmsd, determined for DOX molecules encapsulated in the nanotube interior and (**B**) rmsd of the whole systems both obtained from the last 10 ns of simulations.

Figure 5 completes the conclusions drawn from observation of the MD trajectories by showing how spatial structures of these systems evolve in time. It shows the rmsd of DOX molecules encapsulated in the CNT interior (A) and rmsd of the whole systems (B) determined from the last 10 ns of simulations. As seen in Figure 5A, DOX molecules in the case of E4D20 system, i.e., when corks at the CNT ends have not formed yet, are mobile, and their rmsd fluctuates significantly. This means that DOX tries to escape from the nanotube since there is not much space left within the nanotube. The same analysis in the case of E2D40 and E4D40 systems leads to the conclusion that DOX molecules are almost immobilized in the CNT interior because rmsd is small and flat in both these cases. This effect appeared when other DOX molecules intercalated in iM chains and formed corks at the CNT ends. Thus, the systems E2D40 and E4D40 represent the states with DOX stably entrapped within the carrier structure. On the other hand the system E4D20 might be representative to the case when CNT tips are still open.

However, the systems are not static as seen from the rmsd plots in Figure 5B. They continuously change their spatial structure, and this is related to flanking of iM chains since carbon nanotube and DOX encapsulated within it cannot contribute to the growing rmsd of the whole systems. The most intense increase of rmsd is for the E4D20 system, where iM chains are not zippered by DOX and are flanking freely. The presence of the intercalated DOX reduces flanking of iM chains to some extent, but it seems that they will continue that type of motion, dragging DOX molecules, all the time. Thus, these structures still represent stable nanocontainers for DOX molecules.

The conclusions that can be drawn from the results shown in this section are following: DOX loaded carbon nanotube with iM chains covalently attached to the CNT tips can work as platform for storage of DOX and also ensure good (but not total) isolation of DOX from the environment. The key phenomenon responsible for that property occurs in the area of iM bundles and is directly related to the intercalation of DOX molecules within (one or more) iM chains. This an important new observation since previous studies utilizing carbon nanotubes or other nanoparticles and iM chains required also the complementary guanine rich DNA strands for realization of drugs release from the carrier [16,17,18,19,20,21], and transitions between Watson–Crick pairing to Hoogsteen pairing as the driving force. In this work, we utilize the property of DOX intercalation within one or more iM chains in the form of a random coil and the loss of intercalation when iM’s fold into i-motif structure. Further, we found that intercalated DOX can block other DOX molecules residing in the CNT interior. They can be unblocked when iM folds into i-motif at acidic pH.

### 2.4. Intercalation of DOX in DNA Chains 

Intercalation property of iM chains, as described in previous section, suggests that the role of carbon nanotube is twofold. It can offer a closed volume for storage of DOX molecules, which is very useful, but it can only act as a core for covalent binding of iM chains. In the former case we still need to confirm that DOX can be released to the bulk in response to pH reduction. However, in the latter case we can reduce the role (size) of the nanotube and obtain a new construct with the structure similar to a dendrimer. Let us consider such a new construction in more details. First, the reduction of the CNT size allows for longer simulation times because the number of atoms in the simulation box is significantly smaller (see Table 1 and number of water molecules in relevant cases). Another important remark is that the essential properties or conclusions coming from the analysis of such dendrimer-like systems are directly transferable to the family of systems from Figure 2, Figure 3 and Figure 4. This is because the essential mechanism of DOX binding relies on the intercalation of DOX molecules within the iM chains.

**Figure 6:**
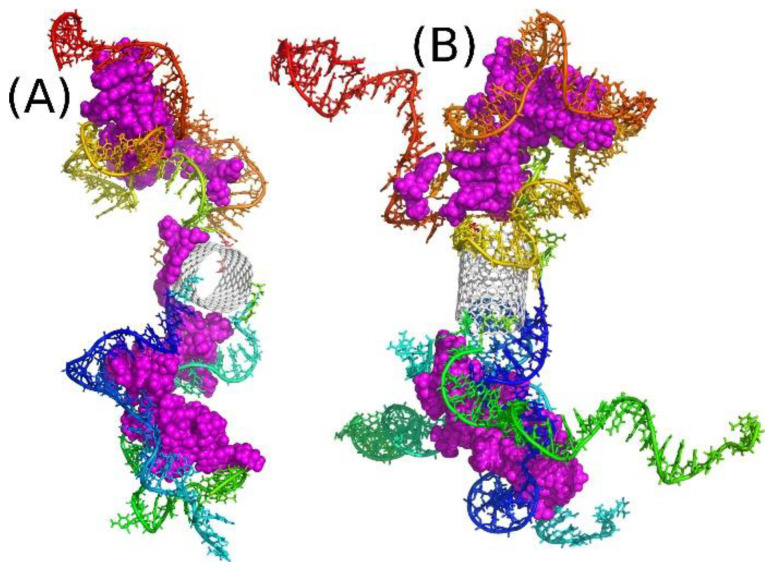
(**A**) The structure of the system I2N after 50 ns of simulations; (**B**) the structure of the system I4N after 50 ns of simulations.

The structures shown in Figure 6 are two examples of such dendrimer-like constructs with two or four iM chains on each side of the carbon nanotube core. These structures were obtained after 50 ns of simulations at the neutral pH, i.e., for the random coil forms of iM chains. As can be seen all DOX molecules were intercalated in the iM chains, but it seems that the capacity of the system I2N was exceeded because one DOX molecule located on the CNT sidewall. In the case of I4N system all drug molecules are entangled in the network of iM chains and none of them was able to escape within the available simulation time. 

**Figure 7:**
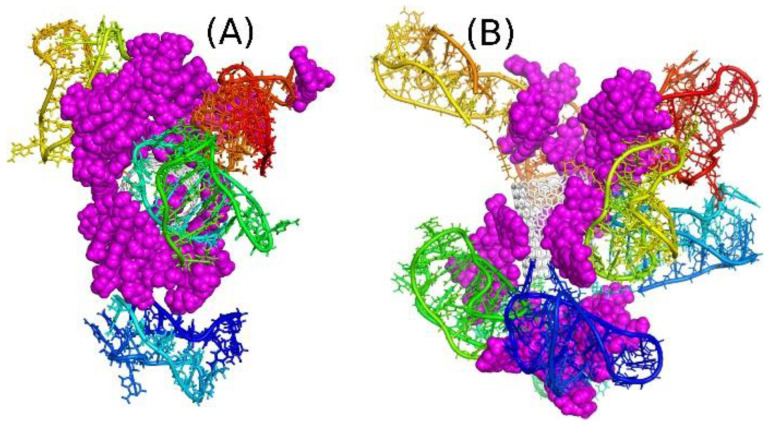
(**A**) The structure of the system I2A after 50 ns of simulations; (**B**) the structure of the system I4A after 50 ns of simulations.

Before analyzing the interaction energies, let us compare geometries of systems I2N and I4N with their counterparts at acidic pH. As the iM chains undergo folding transitions at acidic pH, the systems from Figure 6 experience strong structural changes, as illustrated in Figure 7. As seen, each iM chain folded into an i-motif spatial form, and due to the very compact form of i-motif, the DOX molecules are not able to intercalate between the iM chains. Actually, the channels formed by two or more iM chains in the neutral pH (Figure 6) disappeared, and thus DOX adsorbed only on the ‘external surface’ of the CNT-iM carrier. Moreover, it seems that DOX prefers formation of bigger clusters localized in the center of the carrier close to the nanotube core. Another important conclusion is that in the I2A and I4A systems DOX is more exposed to the solvent, which means that the states I2A and I4A are the states with DOX unlocked and ready to interaction with the target. However, that conclusion needs to be supported by analysis of adequate descriptors like change in intermolecular interaction energy or free energy of binding.

Table 2 shows collection of various components of the intermolecular interaction energies for DOX. The most important information comes from analysis of changes of those energy components when a system transfers from its neutral pH case (N suffix) to the acidic case (suffix A). Therefore, looking at the E_DOX-iM_, we can notice that interaction of DOX with iM chains is stronger at the neutral pH than at the acidic one. The differences in energies are 2137 kJ mol^−^^1^ and 1328 kJ mol^−^^1^ for the I2 and I4 systems, respectively. Additionally, about half of these differences come from either dispersion or electrostatic components. The role of electrostatic interactions is rather obvious: at neutral pH, the iM chains have no protonated cytosines, but at the acid one, each iM chain gets three extra protons, and this reduces the interaction with positively charged DOX. Nevertheless, the role of dispersion interaction components is still very important, as it covers half of the total pair interaction energy. This is because the systems undergo large structural changes leading to lesser contact of DOX with iM after folding it to i-motif structure. These reductions of pair energies are accompanied by the increase of interaction with the CNT cores. Therefore, we can see that DOX significantly loosened its attraction with iM and enhanced with the CNT and solvent. The latter component is very important since it illustrates enhanced solvation of DOX at acidic pH. We can see that the switch from the neutral to acidic pH leads to increase of interaction energy of DOX with solvent by 1024 kJ mol^−^^1^ and 1124 kJ mol^−^^1^ for the I2 and I4 cases, respectively. Similarly, we observe a large increase of solvent accessible surface area (SASA) for DOX when we switch from neutral to acidic pH. All that means that reduction of pH leads to weaker binding of DOX within the carrier structure and we can conclude that the carrier is able to realize the controlled DOX release at acidic pH and stable binding of DOX at the neutral pH. 

Analysis of E_DOX-DOX_ component and N_max_ (number of DOX molecules in the biggest cluster formed) leads to the conclusion that in neutral pH cases DOX molecules form two distinct clusters (one per one side of CNT) and are firmly grouped in those clusters. In the acidic pH cases, the energies of interaction between DOX molecules are reduced, and DOX are grouped mainly into one big cluster, thus the molecules are loosely grouped into that cluster and perhaps they can detach from the carrier due to thermal fluctuations. However, we did not observe spontaneous detachment of drug molecules within the available simulation time.

Attempts to determine the free energies of binding of DOX in the studied systems failed mainly due to the lack of convergence and doubts concerning the reliability of the obtained values. Instead, we can refer to some published data, which can support the central conclusion concerning the release efficacy of the analyzed systems. Therefore, in our previous study we analyzed free energies of binding of DOX to isolated iM molecule either in its random coil form or in the folded i-motif structure [22]. We found that detachment of DOX molecule from the random coil form of iM at the neutral pH is associated with the free energy barrier of the order of 150 kJ mol^−1^ and the detachment at the acidic pH (in i-motif form) is much easier as it needs ca. 40–50 kJ mol^−1^. We can thus assume that the binding of DOX at the neutral pH within the systems I2N and I4N is, at least, not weaker. Thus, it would be enough to store DOX molecules within the carrier for a long time. Switching the pH would lead to about 3-fold weaker binding, by analogy to ref. [22], and ability of spontaneous release of DOX from the I2A and I4A systems. The presence of CNT makes, however, direct comparisons difficult, since CNT contributes to the binding of DOX to the carrier. However, we can expect that this contribution is not decisive, since our earlier studies (also experimental), concerning interaction of DOX with carbon nanotubes, suggested that DOX binds to the surface of CNT weakly. Additionally, without a specific co-adsorbate (Congo Red molecule), the adsorption is unstable [33,34]. We can thus anticipate that the systems I2A and I4A can spontaneously release DOX at acidic pH. 

**Figure 8:**
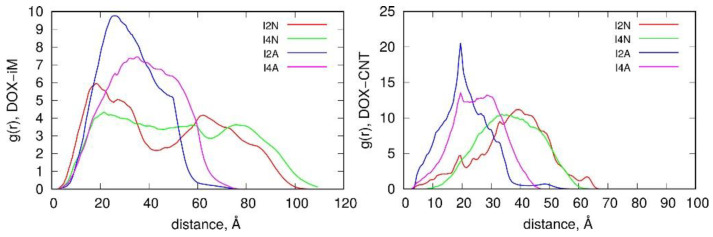
Radial distribution functions, g(r), between DOX center of mass and iM fragments (**left panel**) and between DOX center of mass and CNT (**right panel**).

Figure 8 completes the discussion of interaction energies and structural factors by presenting radial distribution functions, g(r), for DOX—iM pairs and also for DOX-CNT pairs. As both pairs are many-atomic molecules, the distances are computed between center of mass of DOX (which is relatively small molecule) and individual atoms of the second component. Analysis of those functions gives insights into long-time behavior of the systems states. Thus, we can conclude that the switch between neutral form of the carrier and the acidic one is clearly visible in g(r) functions. The upper part of Figure 8 shows that in the systems I2N and I4N DOX reveals two density maxima: one at close contact with iM atoms and second at much larger distance. This is of course related to intercalation of DOX in two sides of the nanotube core within the iM bundles. In the case of I2A and I4A systems the positions of DOX molecules are more fuzzy and shifted to closer distances from iM atoms. This is of course related to unlocking of DOX from iM chains and localization of DOX close to the CNT core.

The g(r) functions of DOX-CNT are shown in the right panel of Figure 8 and they confirm the change in DOX localization within the carrier after transition of iM from random coil to i-motif. Thus, at neutral pH, DOX is localized at large distances from the CNT core, that is in the iM bundles. The systems in acidic pH reveal enhanced probability of DOX adsorption on the CNT core, which confirms unlocking of DOX from the iM bundles.

To sum up, such a dendrimer-like structure, built of a very short CNT fragment and several iM chains, is able to perform reversible swelling and shrinking cycles due to transitions of iM’s between random coil and i-motif spatial structures in response to pH change. Moreover, in the unfolded state, DOX molecules are strongly bound to the carrier due to intercalation of DOX within iM chains. After folding of iM to the i-motif structure, DOX molecules are bound to the carrier much weaker and are able to transfer to the bulk spontaneously.

## 3. Summary and Conclusions

Covalently functionalized carbon nanotubes with iM chains as functional groups revealed promising properties as pH controlled carriers of doxorubicin. We analyzed two mechanisms of locking DOX molecules within the carrier. The first and the most obvious was the encapsulation of DOX in the internal space of carbon nanotube and taking advantage of folding/unfolding transition of iM fragments as gate closing/opening factors. That mechanism turned out to be a slightly different than expected since the iM chains in the random coil state (neutral pH) tend to separate from each other and the nanotube entrance is then fully open. In such a state, DOX molecules were able to diffuse out of the nanotube naturally. Interestingly, the escaping DOX molecules started working as a zipper for iM chains and underwent intercalation within the iM chains. Such a state, with DOX intercalated within iM bundles turned out to be quite effective gate closing factor for other DOX molecules still residing in the CNT interior.

Further studies were focused on the second mechanism that is locking the drug molecules within the iM chains due to previously recognized intercalation mechanism. In such a case we actually treated the CNT as a core for binding the iM chains and obtained a kind of a dendrimer with branches formed by the iM chains. That model of DOX carrier turned out to be interesting since analysis of various energy components led to the conclusion that at the neutral pH DOX molecules should be strongly bound to the carrier but at acidic pH, after folding of iM chains into i-motifs, a significant reduction of the binding energy should be observed. Thus, the mechanism of function of that carrier is similar to dendrimers, which in response to pH change, swell and shrink, and the intercalated drug is either locked or released.

## 4. Methods

The force field type for DNA fragments was the amber ff99 force field for nucleic acids including the bsc1 modifications [35,36]. The force field topology for both native and protonated forms of iM were generated using LEaP program linked with suitable libraries for protonated nucleic acids. 

In the case of amino linker, we used the general amber force field, gaff, due to compatibility with the ff99 force field for nucleic acids. However, gaff needs a proper determination of partial charges, thus we used for that purpose the RESP/ESP charge derive server (https://upjv.q4md-forcefieldtools.org/REDServer-Development (accessed on 22 January 2021)) [37,38]. The quantum chemical computations performed by RESP/ESP server need a small molecule due to performance. Therefore, we used the structure from Figure 1B as it represents the amino linker structure capped by benzoic acid residue and methyl group, which mimic its chemical neighborhood in the end product. 

Carbon nanotube should, in principle, be described using many-body potential; however, we applied a typical molecular approach because of two reasons. First, the mechanical properties of carbon nanotubes are in this case not very important provided that their tubular shapes are well preserved. Second, the applied molecular dynamics software that is gromacs [39] is not compatible with many-body potentials routinely used for carbonaceous materials. It, however, turned out that the molecular approach for carbon nanotubes is in our case fully justified, as it generated sufficiently stiff tubular structures. 

Doxorubicin, DOX, was used in its protonated form because in the considered conditions of pH (5–8) and its pKa = 8.2 the standard unprotonated form is very unlikely [40]. The force field components for DOX were determined according to the same scheme like in the case of amino linker residue. Thus, first the partial charges were determined using RESP server and next the full force field topology was generated using LEaP.

The calculations were done in NPT ensemble using 2 fs integration timestep. All calculations were carried out using TIP3P water model and suitable amounts of Na^+^ and Cl^−^ ions giving 0.145 mol L^−1^ ionic strength of solution. At the very beginning, the solvated systems we subjected to 1000 steps of energy minimization. Next, each system was equilibrated in NVT ensemble at 310 K for 2 ns and in the NPT ensemble for the next 2 ns. The pressure and temperature were set to 310 K and 1 atm, respectively. Interaction cutoff was set to 12 Å and long range electrostatic interactions were computed using particle mesh Ewald scheme. In all equilibration steps, CNT, DOX and iM chains were kept fixed in their initial positions using harmonic restraints, whereas water molecules were allowed to reorganize. The production runs, without position restraints, were carried out in NPT ensemble using 2 fs integration timestep. The periodic boundary conditions were applied in all directions.

## Figures and Tables

**Table 1:** Parameters of the analyzed systems.

Name	CNT Length, Å	Number of DNA Chains on Each Side	pH	Number of DOX Molecules	Number of Water Molecules
E4D20	100	4	neutral	20	248,339
E4D40	100	4	neutral	40	246,544
E2D40	100	2	neutral	40	247,719
I2N	20	2	neutral	30	101,423
I4N	20	4	neutral	30	100,296
I2A	20	2	acidic	30	37,255
I4A	20	4	acidic	30	36,242

**Table 2:** Pair interaction energies (sum of Coulomb and Lennard-Jones contributions) of DOX with other components of the systems, solvent accessible surface area for DOX and number of DOX molecules in the biggest cluster. Energies are expressed in kJ mol^−1^.

System	E_DOX-iM_	E_DOX-CNT_	E_DOX-CNT/iM_	E_DOX-DOX_	E_DOX-Sol_	DOX SASA (Å^2^)	N_max_
I2N	−6169.14 ± 368.39 −4304.12 ± 279.19 *	−332.20 ± 24.05	−6501.34 ± 369.20	−13,613.11 ± 169.29	−9007.70 ± 306.19	7900.33 ± 252.70	15 ± 0.8
I4N	−7160.94 ± 313.70 −5205.18 ± 247.75 *	−7.36 ± 6.92	−7168.29 ± 315.23	−13,880.85 ± 158.42	−7783.71 ± 335.71	6913.92 ± 286.23	15 ± 0.1
I2A	−4031.98 ± 323.11 −3396.64 ± 301.49 *	−1581.86 ± 103.39	−5613.83 ± 328.15	−13,211.97 ± 173.55	−10,031.52 ± 259.08	8551.25 ± 210.79	28 ± 0.2
I4A	−5833.06 ± 238.00 −4427.44 ± 212.68 *	−709.01± 49.42	−6542.12 ± 236.75	−13,067.73 ± 158.67	−8908.34 ± 292.37	8443.85 ± 204.07	21 ± 8.0

* Electrostatic component.

## Data Availability

The data presented in this study are available on request from the corresponding author.
